# Effect of individually tailored nutritional counselling on protein and energy intake among older people receiving home care at risk of or having malnutrition: a non-randomised intervention study

**DOI:** 10.1186/s12877-022-03088-2

**Published:** 2022-05-04

**Authors:** Tarja Kaipainen, Sirpa Hartikainen, Miia Tiihonen, Irma Nykänen

**Affiliations:** 1 Kuopio Research Centre of Geriatric Care, School of Pharmacy, Faculty of Health Sciences, University of Eastern Finland, P.O.B 1627, FI-70211 Kuopio, Finland; 2 Institute of Public Health and Clinical Nutrition, School of Medicine, University of Eastern Finland, P.O.B 1627, FI-70211 Kuopio, Finland

**Keywords:** Cognition, Nutrition, Protein, Clinical trials, Interventions

## Abstract

**Background:**

With ageing, food intake may decrease and lead to an insufficient nutrient intake causing protein-energy malnutrition (PEM) which is associated with adverse health effects and increased mortality. The aim of this study was to investigate the effects of individually tailored dietary counseling focused on protein intake among home care clients with PEM or at risk of developing PEM. The secondary aim was to study the intake of energy and other nutrients.

**Methods:**

This intervention study is part of the non-randomised population-based multidisciplinary Nutrition, Oral Health and Medication study (NutOrMed study). The intervention group comprised 112 and the control group 87 home care clients (≥75 years) with PEM or risk of PEM. PEM was defined by Mini Nutritional Assessment score < 24 and/or plasma albumin < 35 g/L. The nutrients intake was assessed from 24-hour dietary recall at the baseline and after the six-month intervention. The intervention consisted of an individually tailored dietary counseling; the persons were instructed to increase their food intake with protein and energy dense food items, the number of meals and consumption of protein-, energy- and nutrient-rich snacks for six months.

**Results:**

After the six-month nutritional intervention, the mean change in protein intake increased 0.04 g/kgBW (95% CI 0.05 to 0.2), fibre 0.8 g (95% CI 0.2 to 4.3), vitamin D 8.5 μg (95% CI 0.7 to 4.4), E 0.6 mg (95% CI 0.4 to 2.2), B12 0.7 μg (95% CI 0.02 to 2.6), folate 8.7 μg (95% CI 1.5 to 46.5), iron 0.4 mg 95% CI 0.6 to 2.4), and zinc 0.5 mg (95% CI 0.6 to 2.2) in the intervention group compared with the control group**.** The proportion of those receiving less than 1.0 g/kg/BW protein decreased from 67 to 51% in the intervention group and from 84 to 76% in the control group. Among home care clients with a cognitive decline (MMSE< 18), protein intake increased in the intervention group by 0.2 g/kg/BW (*p* = 0.048) but there was no change in the control group.

**Conclusion:**

An individual tailored nutritional intervention improves the intake of protein and other nutrients among vulnerable home care clients with PEM or its risk and in persons with cognitive decline.

**Trial registration:**

ClinicalTrials.gov: NCT02214758. Date of trial registration: 12/08/2014.

## Background

An increasing proportion of older population are encouraged to live in their own homes with the help of home care [1, 2]. This shift from residential care to home care means that home care clients are more vulnerable with several comorbidities [2]. In Finland, municipalities and private organizations provide home care including home help, nursing with treatments and administering medications and medical care services.

Food intake often decreases with ageing due to diseases and changes in appetite, and these might lead to an insufficient intake of nutrients, such as protein, energy, fibre and micronutrients [3–7]. When energy and protein intake are inadequate, this leads to a condition termed protein-energy malnutrition (PEM) [1]. Previous studies have found that PEM is associated with a reduced physical function and a poorer quality of life [8] as well adverse health events, an increased risk of falls and even increased mortality [9–12]. These adverse effects can compromise their independence [13, 14]. One of the challenges in the provision of home care for older clients is to take care of their nutritional status to maintain their health and wellbeing [10]. This is especially challenging among older vulnerable people suffering from a cognitive decline [15].

Our previous study found that it is possible to improve the nutritional status of home care clients by initiating an intervention focused on individual nutritional counseling [16]. Previous nutritional interventions studies in residential care among persons with PEM or its risk have demonstrated that it is possible to increase protein and energy intake of older vulnerable persons [17]. To our knowledge, there are no previous studies which have evaluated the effect of nutritional interventions in older home care clients with PEM or at its risk by improving their intake of nutrients. The aim of this study was to investigate the effects of individually tailored dietary counseling focused on protein intake among home care clients with PEM or its risk. The secondary aim was to study the intake of energy and other nutrients.

## Methods

### Design and participants

**Fig. 1:**
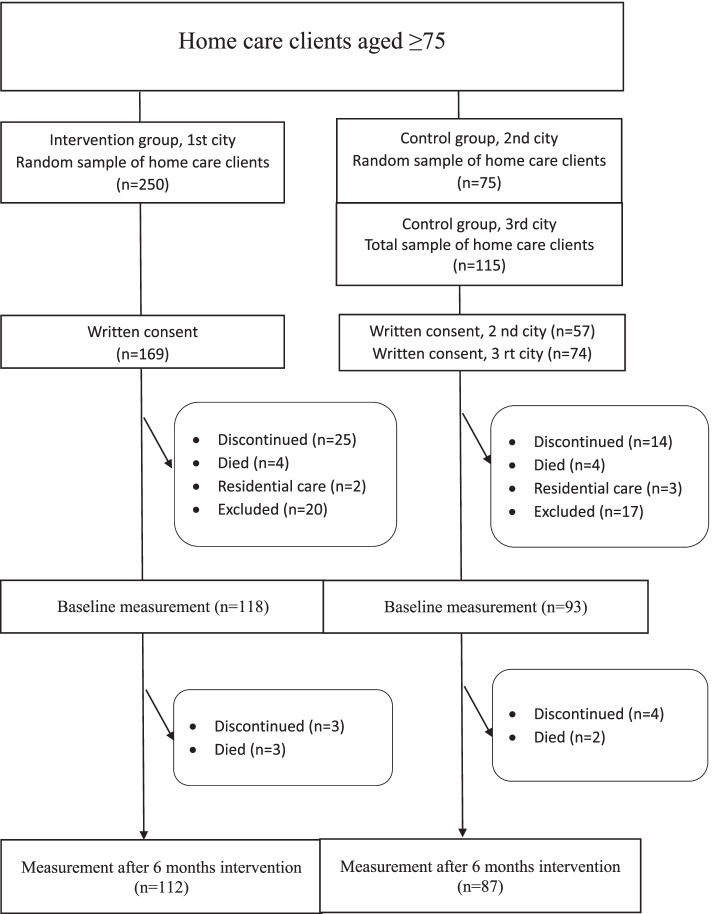
Flowchart of the stydu

This intervention study is part of the non-randomised population-based multidisciplinary Nutrition, Oral Health and Medication study (NutOrMed study) aimed at evaluating nutritional status, oral health, functional ability and hospitalization use and costs among home care clients. NutOrMed study was carried out from 2013 to 2014. The NutOrMed study sample consisted of home care clients aged 75 to 99 years living in three cities in Eastern and Central Finland and who had regular home care, i.e. home care at least once a week. The intervention group was a random sample of 250 home care clients and a control group of 190 home care clients (Fig. 1). The intervention city was big enough to allow us to get a random sample, but the other two towns were smaller, and both towns were needed as control groups to maximise the number of controls. For the same reason, all the homecare clients in community III had the possibility to participate (total sample). To avoid contamination, the intervention group was situated approximately 100 km away from the towns of the control groups. Randomisation inside communities I and II was done with a coded list of homecare clients and an SPSS random sample tool. The study was introduced to the persons included in the sample by home care nurses both verbally and with a written bulletin. After that, those willing to participate gave their written consent [18].

The population (*n* = 236) of this study consisted of home care clients who completed the MNA test and 24-hour dietary recall, and the intervention was implemented for those who were at PEM or risk of PEM. After exclusion of those persons in good nutritional status (*n* = 37), the PEM intervention group consisted of 112 and the control group of 87 participants. We had no exclusion criteria regarding age, morbidity, or cognitive status. If the participant was unable to reply, the data was supplemented by a caregiver or his/her own nurse. More details on the NutOrMed study have been described in a previous study [18].

### Nutritional intervention

The nutritional intervention was tailored based on the baseline MNA test, plasma albumin and 24-hour dietary recall. The definition for PEM or the risk for PEM was MNA score < 24 and/or plasma albumin < 35 g/l (Table 1). For those with PEM or its risk a clinical nutritionist provided one nutritional counseling session at the baseline based on 24-hour dietary recall. At the same visit a clinical nutritionist planned a personal nutritional care together with the client and her/his nurse or family members. 24-hour dietary recall was analysed at the same visit based on national nutritional recommendations for the amounts used by different food groups and participants weight to get enough protein and energy. The nutritional care plan was based on an international recommendation on protein intake [10] and national nutrition recommendations for the older persons [19]. The target for protein intake was 1.0 g/kg BW and energy intake 30 kcal/kg per day [10]. The assessment of nutritional status by a clinical nutritionist was done at baseline and after the six-month intervention in both study groups.

**Table 1:** Description of the procedures in the intervention and control group

Home visits	Procedures in the intervention group	Procedures in the control group
1st home visit (a clinical nutritionist) Intervention continued by homecare nurses and family caretakes after a clinical nutritionist home visit.	Baseline measurements: • examined weight, height and daily eating routines with 24-hour dietary recalls • collected history of health problems, food preferences and appetite status • evaluated nutritional status with the MNA test and plasma albumin and nutrient intake by using the 24-hour dietary recalls Individual tailored nutritional care plan: • increased their protein and energy intake with o protein^1^- and energy^2^ dense food items o the number of meals o consumption of energy-, protein- and nutrient-rich snacks • used daily vitamin D (20-μg) supplementation • advised on other food-related issues such as o grocery shopping and cooking o appetite o eating-related problems • handed special food-related leaflets covering increasing protein and energy intake and booklet of good nutrition for older adults	Baseline measurements: • examined the client’s weight, height and daily eating routines with 24-hour dietary recalls • collected the client’s history of health problems, food preferences and appetite status • evaluated the client’s nutritional status with the MNA test and plasma albumin and nutrient intake by using the 24-hour dietary recalls Not received intervention.
2nd home visit after 6 months (a clinical nutritionist)	Re-examined measurements: • examined weight, height and daily eating routines with 24-hour dietary recalls • collected history of health problems, food preferences and appetite status • evaluated nutritional status with the MNA test and plasma albumin and nutrient intake by using the 24-hour dietary recalls • if needed repetition of received instructions on how to follow the given nutritional care plan (clients, their nurse or family members)	Re-examined measurements: • examined the client’s weight, height and daily eating routines with 24-hour dietary recalls • collected the client’s history of health problems, food preferences and appetite status • evaluated the client’s nutritional status with the MNA test and plasma albumin and nutrient intake by using the 24-hour dietary recalls • booklet of good nutrition for older adults

*MNA* Mini Nutritional Assessment; 1. To increase protein intake: use plenty of dairy products; boiled in milk porridge, cheese on bread, snack with cheese, yogurt or milk, foods milk powder, high protein dairy products, and hot meal with meat, fish or eggs; 2. To increase energy intake: eat several small meals a day e.g. oil to foods, margarine on a slice of bread.

The intervention was intended to increase food intake of energy-dense foods, the number of meals and their consumption of energy-, protein- and nutrient-rich snacks, such as slices of bread with margarine, yogurt and curd cheese. If the participant had a lack of energy, she/he was advised to eat small meals more frequently during the day and to increase the use of vegetable oil in foods and/or margarine on a slice of bread and use fat dairy products. If the participants diet did not include enough protein for her/his needs, she/he was advised to consume more dairy products like milk as a drink, cheese on bread, snack with curd cheese, quark, yogurt, curdled milk and cottage cheese and boiled porridge made with milk. In addition, participants were advised to use of milk powder in foods and high protein dairy products and to eat daily two warm meals with meat, chicken, fish or eggs. According to guidelines we recommended the use of a vitamin D supplementation of 20/g/day [19], but these were excluded from in the analysis. Only the dietary intake of vitamin D was included in the analyses. In this intervention did not prescribe multivitamin supplements to the participants.

### Data collection

#### Outcome measurements

A clinical nutritionist assessed nutrient intake with 24-hour dietary recall at the baseline and after the six-month follow-up in both groups. The participants’ nutrients were calculated from the 24-hour dietary recall using AivoDiet program, developed for nutrient counting [20]. We compared protein intake with the recommendations in the ESPEN guidelines i.e. a protein intake should be at least 1 g/kg/BW per day, adjusted for individual needs [10].

#### Characteristics and covariates

All participants were interviewed and examined at home by trained nurses, a clinical nutritionist, dental hygienists and a pharmacist. The participants´ nutritional status was assessed with Mini Nutritional Assessment (MNA) by a clinical nutritionist. The MNA is a validated and standardized tool for detecting the nutritional status of older people [21–24]. Body weight was measured to the nearest 0.1 kg by a beam scale with the subject wearing light clothes and without shoes. Height was measured standing, the head in the Frankfurt Plane position. If the participant was unable to stand, height was measured using indirect demi-span methods. Demi-span is the distance from the midline at the sternal notch to the web between the middle and ring fingers along outstretched arm [25, 26]. After that, height was calculated by a standard formula [25]. Plasma albumin levels were measured according to standard protocols at the regional laboratory, ISLAB [27].

Oral health was assessed by a dental hygienist. Dry mouth was assessed by asking the participants “Do you have a feeling of a dry mouth?” The question had three categories from none to continuously. Categories 2 and 3 (occasionally and continuously) were combined in the analyses. Chewing problems were assessed by asking the participants, “Do you have chewing problems?” The question had two categories: “yes” and “no”. A pharmacist recorded each prescription and over-the-counter drugs being used regularly as well as those on an as needed based on the interview, medication lists and medication packages at the baseline.

Comorbidity was defined using a modified version of the Functional Comorbidity Index (FCI) [28, 29]. The diagnosis of any cognitive disorder was based on medical records with the diagnosis being verified by a geriatrician. Depressive symptoms were assessed with the 15-item Geriatric Depression Scale (GDS-15) and cut-off was ≥5 [30]. Functioning was assessed by Activities of Daily Living (ADL) [31] and Instrumental Activities of Daily Living (IADL) [32] and cognition was assessed by the Mini Mental State Examination (MMSE) [33]. Self-reported ability to walk 400 m was assessed by asking the participants “Can you walk at least 400 meters?” The question had four response categories: 0 (unable to walk), 1 (unable to walk independently) and 2 or 3 (able to walk independently with or without difficulties). Categories 2 and 3 were combined for the analyses. The baseline characteristics included demographic data. All the measurements were performed at the baseline and after the six-month follow-up, except for the drug use and comorbidities which were evaluated only at the baseline.

### Statistical analysis

Statistical comparisons between the characteristics of the two groups were made using the independent t-test or the chi-square test when appropriate. A general linear model univariate analysis was adopted to compare the effect of the intervention between the groups adjusted for age, gender, education years, cognitive decline, and baseline nutrient. Participants assessed at baseline and six months were included in the data analysis (per protocol). *P*-values < 0.05 were considered significant. The data were analyzed using SPSS 27.0 software.

## Results

At baseline, the mean age of participants was 84.3 years in both groups, the majority i.e. 72% were female (Table 2). The participants in the intervention group had more years of education, and lower values of BMI, FCI and albumin compared to the control group. There were no significant differences in cognition and functional abilities between the groups.

**Table 2:** Baseline characteristics of participants with protein-energy malnutrition or risk of it in the intervention and control group

	Intervention group (*n* = 112)	Control group (*n* = 87)	P-value
Demographic			
Female, % (n)	73.2 (82)	71.3 (62)	0.760
Age, mean (SD)	84.3 (5.1)	84.3 (5.3)	0.983
Education in years, mean (SD)	9.0 (3.9)	6.8 (1.8)	< 0.001
Living alone, % (n)	65.5 (72)	66.3 (57)	0.904
Clinical			
MNA, mean (SD) (range)	21.2 (2.1) (12.5–27.5)	21.6 (2.3) (12.5–27)	0.274
≥24% (n)	87.5 (98)	87.4 (76)	0,977
≤23.5% (n)	12.5 (14)	12.6 (11)	
BMI (kg/m^2^), mean (SD) (range)	26.6 (5.5) (15.6–46.1)	28.4 (6.4) (17.7–51.8)	0.043
< 24% (n)	27.6 (31)	22.9 (20)	0.053
24–29% (n)	47.3 (53)	40.2 (35)	
> 29%(n)	35.0 (28)	36.8 (32)	
Plasma albumin **(g/L)**, mean (SD) (range)	35.3 (3.0) (27.0–40.0)	36.8 (3.8) (28.0–45.0)	0.028
Number of drugs ≥10, % (n)	53.2 (59)	60.9 (53)	0.274
Oral health			
Dry mouth, % (n)	57.7 (64)	56.3 (49)	0.851
Chewing problems, % (n)	18.8 (19)	19.8 (17)	0.896
Functional clinical			
FCI, mean (SD)	2.5 (1.7)	3.6 (2.0)	< 0.001
cardiovascular diseases, % (n)	61.6 (69)	65.5 (57)	0.570
diabetes, % (n)	29.5 (33)	34.5 (30)	0.450
MMSE, mean (SD)	23.1 (5.3)	22.6 (5.1)	0.554
< 24, % (n)	40.7 (44)	47.6 (40)	0.341
< 18, % (n)	13.9 (15)	14.3 (12)	0.937
GDS-15, ≥5, % (n)	44 (48)	52.9 (46)	0.219
Functional ability			
ADL, mean (SD)	83.1 (18.2)	84.9 (20.0)	0.512
< 60, % (n)	11.0 (12)	8.1 (7)	0.502
IADL mean (SD)	4.8 (2.2)	4.4 (2.3)	0.249
< 5, % (n)	43.1 (47)	50 (42)	0.342
Walks 400 m independently, % (n)	60 (66)	60.9 (53)	0.896

*SD* Standard deviation, *MNA* Mini Nutritional Assessment, *BMI* Body Mass Index, *FCI* Functional comorbidity index, *MMSE* Mini Mental State Examination, *GDS-15* Geriatric Depression Scale-15, *ADL* Activities of Daily Living (Barthel Index), *IADL* Instrumental Activities of Daily Living (Lawton-Brody).

At the baseline among all participants, the mean intake of protein and energy was higher in the intervention group compared to the control group (Table 3). The mean daily energy intake was 22.3 (kcal/kgBW) in the intervention group and 18.1 (kcal/kgBW) in the control group. Energy intake was less than 30 (kcal/kgBW) in 67.5% of the participants in the intervention group and 89.1% in the control group. The mean energy intake increased in both groups, but the difference was not significant between groups.

**Table 3:** Dietary intake of nutrients at baseline and after intervention among homecare clients with protein-energy malnutrition or risk of it in the intervention and control group

	Intervention group (*n* = 112)		Control group (*n* = 87)		Difference between groups Δ6th month		
	Baseline, mean (SD)	Change ≥6 mo, mean (SD)	Baseline, mean (SD)	Change ≥6 mo, mean (SD)	Mean	95% CI	*P* value
Energy **(kcal/d)**	1490.3 (415.7)	15.0 (379.9)	1278.9.0 (377.4)	92.8 (441.8)	−77.8	−33.7 to 182.0	0.177
Energy (kcal/kgBW)	22.5 (7.3)	0.2 (5.8)	17.7 (5,6)	1.4 (6.3)	−1.2	−3.0 to 0.5	0.158
Carbohydrates (kcal/d)	723.8 (224.1)	−5.6 (207.0)	644.8 (198.5)	48.7 (249.1)	−54.3	−118.1 to 9.4	0.094
Fat (kcal/d)	468.6 (167.1)	−8.6 (187.1))	379.8 (152.8)	24.5 (173.0)	−33.1	−84.1 to 17.9	0.202
Protein (kcal/d)	239.2 (72.4)	24.5 (76.3)	210.4 (67.4)	13.2 (75.8)	11.3	−10.1 to 32.7	0.289
Carbohydrates E%	49.3 (6.5)	−0.8 (8.1)	50.6 (7.5)	−0.1 (9.0)	−0.7	−3.0 to 1.7	0.577
Fat E%	31.3 (5.7)	−0.8 (7.4)	29.4 (6.2)	0.1 (8.4)	−0.9	−3.2 to 1.2	0.383
Protein E%	16.1 (2.8)	1.4 (3.7)	16.5 (3.1)	−0.3 (3.9)	1.7	0.6 to 2.7	0.002
Protein (g/kgBW)	0.9 (0.3)	0.09 (0.3)	0.7 (0.2)	0.05 (0.3)	0.04	0.05 to 0.2	0.003
Fibre (g)	20.7 (8.7)	0.8 (8.4)	18.3 (6.9)	−0.3 (6.3)	1.1	0.2 to 4.3	0.034
Vitamins/d							
A (μg)	651.1 (1103.6)	−45.7 (1133.9)	408.7 (385.2)	134.0 (792.0)	179.9	− 129.6 to 203.8	0.661
D (μg)	8.5 (4.4)	2.1 (8.0)	7.6 (4.0)	0.7 (4.5)	1.4	0.7 to 4.4	0.007
E (mg)	7.8 (3.5)	0.6 (3.6)	6.0 (2.7)	0.5 (2.5)	0.1	0.4 to 2.2	0.004
Thiamine (mg)	1.3 (0.6)	0.02 (0.6)	1.2 (0.5)	−0.03 (0.6)	0.05	0.02 to 0.3	0.024
Riboflavin (mg)	1.8 (0.6)	0.1 (0.6)	1.6 (0.6)	0.2 (0.7)	−0.1	−0.06 to 0.3	0.192
B12 (μg)	3.8 (4.1)	0.7 (5.7)	3.1 (1.9)	0.5 (3.1)	0.2	0.02 to 2.6	0.023
Folate (μg)	210.9 (114.0)	8.7 (107.0)	182.4 (68.4)	8.4 (95.6)	0.3	1.5 to 46.5	0.036
Minerals/d (mg)							
Calcium	979.3 (344.0)	100.0 (380.1)	837.6 (365.7)	113.3 (414.8)	−13.3	−34.4 to 188.3	0.174
Iron	10.0 (3.9)	0.4 (4.0)	8.8 (2.9)	−0.1 (3.0)	0.5	0.6 to 2.4	0.002
Zinc	10.0 (3.1)	0.5 (2.8)	8.9 (2.9)	−0.2 (3.0)	0.7	0.6 to 2.2	0.001

*SD* standard deviation, *CI* confidence interval, *kcal/d* kilocalorie per day, *g/kgBW* gram per kilogram body weight, *g* gram, *μg* microgram, *mg* milligram, *E%* energy per cent, *vitamins/d* vitamins per day.

**Fig. 2:**
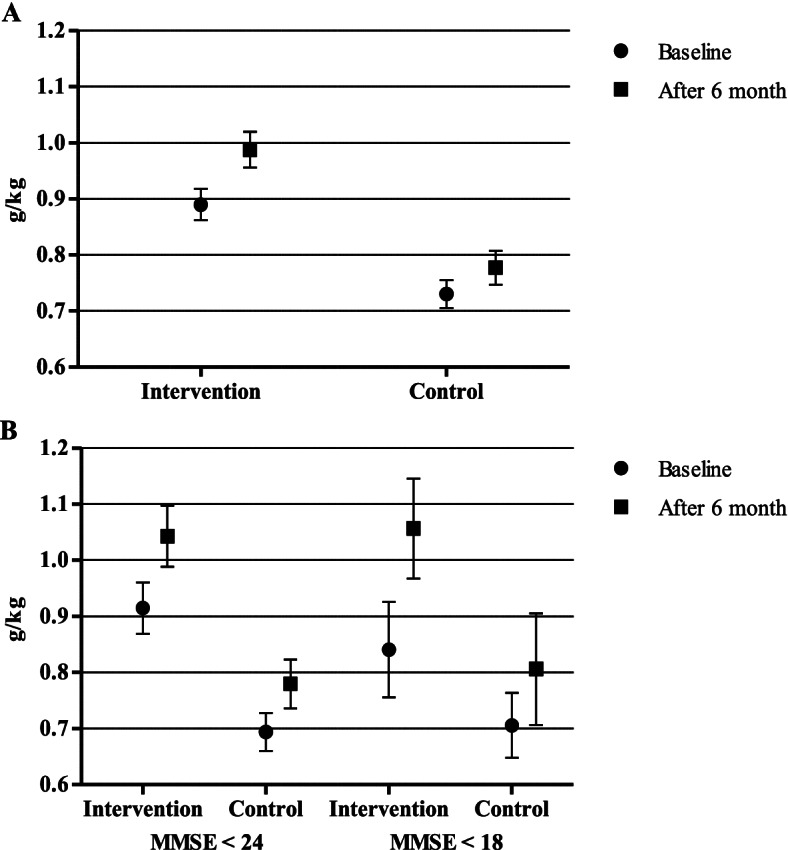
Protein intake at baseline and after 6 months among all participants (**A**). Protein intake at baseline and after 6 months among participants with MMSE score below 24 and 18 (**B**)

The mean protein intake was 0.9 (g/kgBW) in the intervention group and 0.7 (g/kgBW) in the control group (Fig. 2A). Protein intake was less than 1.0 g/kg body weight in 67.9% of participants in the intervention and in 87.2% in the control group. The mean energy intake in the intervention group was 1490 kcal and 1280 kcal in the control group. After the six-month nutritional intervention, the mean change in protein intake increased 0.04 g/kgBW (95% CI 0.05 to 0.2), fibre 0.8 g (95% CI 0.2 to 4.3), vitamin D 8.5 μg (95% CI 0.7 to 4.4), E 0.6 mg (95% CI 0.4 to 2.2), B12 0.7 μg (95% CI 0.02 to 2.6), folate 8.7 μg (95% CI 1.5 to 46.5), iron 0.4 mg 95% CI 0.6 to 2.4), and zinc 0.5 mg (95% CI 0.6 to 2.2) in the intervention group compared with the control group (Table 3).

In the population having an MMSE score lower than 24, the intervention increased protein intake by 0.04 (g/kgBW) in the intervention group as compared to the control group (Fig. 2B). The proportion of participants with a protein intake less than 1.0 g/kgBW declined from 67.2 to 50.8%, but only marginally in the control group i.e. from 83.7 to 76.6%. Among home care clients with cognitive decline (MMSE< 18), in the intervention group, the protein intake increased by 0.2 (g/kgBW) (*p* = 0.048) but there was no change in the control group.

## Discussion

As far as we are aware, this is the first study to show that a six-month individually tailored nutritional intervention was able to improve the intake of protein and other nutrients like fibre, vitamin D, thiamine, vitamin B12, iron and zinc among vulnerable home care clients with PEM or at its risk. Among persons with a cognitive decline, protein intake increased more than in persons with intact cognition.

A previous study observed an increase in the total protein intake among care home residents with PEM or who were at risk of developing PEM [17]. The difference in our study is how the results were estimated and thus it is somewhat difficult to compare the results of these two studies. For example, in the study of Stowe et al. [17], protein intake was expressed as mean grams whereas we measured protein intake as g/kgBW which is in accordance with recommendations and thus can be utilized in clinical work. The clinical significance of the intervention was that protein intake increased thought recommend level of ESPEN was not reached [10]. This is important notice to that these changes were greater in the intervention group than in the control group. It is common in the intervention studies that also control group improves as a part of the participation bias to intervention [34]. This is clearly shown in the previous study [35]. Among older adults either with PEM or at risk of developing PEM, increased protein intake can prevent the associated adverse health consequences and help maintain activities of daily living and thus preserve independence and longer living at home [9–12, 36].

There was no significant change in total energy intake between groups. While protein increased in the intervention group, carbohydrates and fats even decreased slightly. This explains why total energy intake did not increase. The guidance emphasized the consumption of protein-rich foods. In the intervention, the subjects were instructed to consume low-fat or lean meat and dairy products in accordance with Finnish nutritional recommendations [19]. This was also supported by the fact that more than 60% of the subjects had cardiovascular disease.

However, it should be noted, that the energy intake was below the recommended 30 kcal / kgBW [10]. This can lead to amino acids being used for energy-producing reactions. However, if the weight remains stable, as in this study, energy intake and consumption are in balance. Regular weight monitoring is a prerequisite for assessing energy adequacy [19]. In addition, the energy consumption of an older person can sometimes be very low [37].

It was observed that it was possible to increase the protein intake in home care clients with a cognitive decline (MMSE score under 24) e.g., the proportion of those subjects with a protein intake less than 1.0 g/kgBW decreased. This is in accordance with a previous study where a tailored nutritional counseling was provided to home-dwelling older adults with Alzheimer’s disease and their caregivers [38]. That is an important finding because the cognitive decline may decrease the patient’s intake of nutrients due the problems with food preparation, forgetting to eat, loss of appetite and eating problems due to poor oral health [15, 39, 40]. An explanation for the finding in the present study was that the counseling was given not only to family caretakers as in the previous study but also to homecare nurses who were provided with the appropriate counseling information which allowed them to supervise the continuation of the intervention according to the instructions given by a clinical nutritionist. The fact that the intervention was implemented by both homecare nurses and family caretakers was the keystone in its success in persons with cognitive decline.

Our study is the first to evaluate the intake of many important nutrients such fibre, vitamin D, thiamine, vitamin B12, iron and zinc among home care clients with PEM; the intervention improved also their intake as compared to the control group. In our intervention, a clinical nutritionist recommended snacks, such as a slice of bread with margarine, yogurt and curd cheese, porridge, vegetable oil/fats when making food or spreading margarine on a slice of bread, as well ensuring regular consumption dairy and meat products. These were recommended because in the Finnish diet, cereal products are important sources of fibre, iron, zinc and thiamine, meat is a good source of thiamine, vitamin B12, iron and zinc, dairy products provide vitamin D and B12, zinc and vegetable fats are important sources of vitamins D and E [41].

Nutritional counseling in our study was based on normal food items considering the person’s preferences and this may have had a beneficial effect on protein intake**.** If the client’s diet was low in protein, the clinical nutritionist recommended eating two hot meals a day with meat, chicken, fish or eggs and eating plenty of dairy products, which is one of the main sources of protein in Finnish food culture [41].

The result is in line with the ESPEN recommendation, where oral nutrition should always be the first choice i.e. oral nutrition incorporates the sensations of taste and flavor; in other words, nutrition should be a pleasurable experience promoting well-being not simply an intake of nutrients [10].

The strength of this study is that it is a real-life intervention among home care clients without any exclusion criteria regarding maximum age, morbidity, or cognition, and so the population represent real life home care clients. Our findings have potential to be generalized for older people receiving home care with the same kind of home care provision.

We also adopted a multidisciplinary approach with a large number of validated instruments, and population-based planning. An important issue was that the nutritional intervention was individually tailored and based on the participants` food preferences so dietary counseling was probably better accepted by the participants. The reliability of the intervention was improved by having just one clinical nutritionist. We obtained comprehensive data on nutrient intake according to the 24-hour dietary recall that was collected by a clinical nutritionist. In addition, information from persons with a cognitive decline was collected also from family caretakers and homecare nurses.

Our study has some limitations. Those with or at risk for PEM were a subgroup of the entire NutOrMed study intervention group, and thus participants in this study could not be randomised. Second limitation of this study is that data were collected by several nurses, which could impact internal reliability. However, all nurses were trained by the same registered clinical nutritionists. One limitation relates to a 24-hour dietary recall as possible biases due to cognitive problems, perception of food portion sizes, and conceptualization [42]. However, to overcome this prejudice, participants with cognitive impairment were accompanied by a caregiver or nurse (who was familiar with eating habits) during the interview. In addition, during the interview the clinical nutritionist used the Food Portion Picture Book. Food diaries are also not helpful for the elderly with multiple illnesses, weaknesses, vision problems, or people with even mild cognitive impairment. All of these are very common among older home care clients or even criteria for receiving home care in Finland.

In this study the participants in the intervention group had more years of education. A higher level of education may explain better nutrition status [43]. For this reason, in this study, the results were adjusted for the years of education**.**

## Conclusions

An individually tailored nutritional intervention can improve the intake of protein and other nutrients among vulnerable home care clients with PEM, those at risk of developing PEM and also in persons with a cognitive decline. This study shows possibilities to improve nutritional status in older vulnerable home care clients by individual based nutritional counselling.

## Data Availability

The datasets generated and/or analysed during the current study are not publicly available due to limitations of ethical approval involving the patient data and anonymity but are available from the corresponding author on reasonable request.

## Abbreviations

ADL: Activities of Daily Living
BMI: Body mass index
CGA: Comprehensive Geriatric Assessment
CI: Confidence interval
COPD: Chronic obstructive pulmonary disease
FCI: Functional Comorbidity Index
GDS-15: 15-item Geriatric Depression Scale
IADL: Instrumental Activities of Daily Living
MMSE: Mini-Mental State Examination
MNA: Mini Nutritional Assessment
NutOrMed: Nutrition, Oral Health and Medication study
OR: Odds ratio
PEM: Protein-energy malnutrition
SD: Standard deviation
